# The Posterior Signaling Center Is an Important Microenvironment for Homeostasis of the *Drosophila* Lymph Gland

**DOI:** 10.3389/fcell.2020.00382

**Published:** 2020-05-21

**Authors:** Fangzhou Luo, Shichao Yu, Li Hua Jin

**Affiliations:** Department of Genetics, College of Life Sciences, Northeast Forestry University, Harbin, China

**Keywords:** *Drosophila*, lymph gland, posterior signaling center, hematopoietic stem cells niche, differentiation, signaling network, immune response, leukemia

## Abstract

Hematopoiesis is a necessary process for development and immune defense in *Drosophila* from the embryonic period to adulthood. There are two main stages in this process: the first stage occurs in the head mesoderm during the embryonic stage, and the second occurs in a specialized hematopoietic organ along the dorsal vessel, the lymph gland, during the larval stage. The lymph gland consists of paired lobes, each of which has distinct regions: the cortical zone (CZ), which contains mature hemocytes; the medullary zone (MZ), which contains hematopoietic progenitors; and the posterior signaling center (PSC), which specifically expresses the early B-cell factor (EBF) transcription factor Collier (Col) and the HOX factor Antennapedia (Antp) to form a microenvironment similar to that of the mammalian bone marrow hematopoietic stem cell niche. The PSC plays a key role in regulating hematopoietic progenitor differentiation. Moreover, the PSC contributes to the cellular immune response to wasp parasitism triggered by elevated ROS levels. Two recent studies have revealed that hematopoietic progenitor maintenance is directly regulated by Col expressed in the MZ and is independent of the PSC, challenging the traditional model. In this review, we summarize the regulatory networks of PSC cell proliferation, the controversy regarding PSC-mediated regulation of hematopoietic progenitor differentiation, and the wasp egg infection response. In addition, we discuss why the PSC is an ideal model for investigating mammalian hematopoietic stem cell niches and leukemia.

## Introduction

Hematopoiesis in *Drosophila* and vertebrates is highly conserved, and there are significant similarities in the molecular mechanisms between the cardiogenic mesoderm in *Drosophila* and the aorta-gonadal-mesonephros mesoderm in mammals (Medvinsky and Dzierzak, 1996; Mandal et al., 2004). *Drosophila* hemocytes are needed for immune defense, wound healing, tissue integrity and environmental stress responses. Because of the convenience of genetic manipulation, *Drosophila* are regarded as ideal models for research on the regulatory factors of hematopoiesis and the molecular mechanisms of leukemia (Robertson et al., 1988; Crozatier and Vincent, 2011; Baril et al., 2017).

*Drosophila* undergo two main waves of hematopoiesis (Holz et al., 2003). The first occurs in the head mesoderm at the early embryonic stage (Tepass et al., 1994). The promonocytes of this stage can differentiate into mature hemocytes that contribute to populations of sessile hemocytes attached to the cuticle and circulating hemocytes in the hemolymph at the larval stage (Lebestky et al., 2000; Kurucz et al., 2007; Honti et al., 2010). The cardiogenic mesoderm of the embryo subsequently becomes a specialized organ, the lymph gland, and another hematopoietic pool is created in the larval stage (Shrestha and Gateff, 1982; Jung et al., 2005). During metamorphosis, hematopoietic progenitors of the lymph gland differentiate into mature hemocytes and enter the circulation due to dispersal of the lymph gland (Lanot et al., 2001; Grigorian et al., 2011). The hemocytes generated from these two phases can persist into the adult stage. Moreover, a recent study has revealed four clusters of hemocytes located in the dorsal part of the adult fly abdomen, termed “adult hematopoietic hubs” (Ghosh et al., 2015). Three types of terminally differentiated cells are generated by hematopoietic progenitors (Evans et al., 2003). Plasmatocytes are the most numerous circulating hemocytes, representing 90–95% of hemocytes in circulation, and have the ability to phagocytose invading pathogens (Tepass et al., 1994; Evans et al., 2003; Jung et al., 2005). Crystal cells, so named because they contain crystalline inclusions, are other mature hemocytes (Shrestha and Gateff, 1982; Lanot et al., 2001) that can cause melanization reactions for wound healing (Rizki and Rizki, 1990; Rämet et al., 2002). The third type of mature hemocyte is the lamellocyte; these cells are rarely found in healthy larvae (Lanot et al., 2001). The main function of lamellocytes is to encapsulate foreign objects that are too large to be phagocytosed by plasmatocytes (Rizki and Rizki, 1992).

In the third larval stage, the *Drosophila* lymph gland matures and can be separated into three distinct zones: the cortical zone (CZ), the medullary zone (MZ) and the posterior signaling center (PSC) (Jung et al., 2005). Under normal conditions, the CZ consists of mature hemocytes, including plasmatocytes and crystal cells, whereas the MZ, located in the inner region of each lymph gland lobe, contains hematopoietic progenitors that can differentiate into mature hemocytes. The PSC comprises 30∼40 cells at the posterior tip of each primary lobe, acting as a hematopoietic niche (Mandal et al., 2007) similar to the hematopoietic niche in the bone marrow of mammals (Krzemień et al., 2007), and plays a key role in regulating progenitor homeostasis (see section “The PSC Functions as a Hematopoietic Progenitor Niche”). PSC cells are distinguished by the expression of the Notch ligand Serrate (Ser), the early B-cell factor (EBF) Collier (Col) and the HOX factor Antennapedia (Antp) (Lebestky et al., 2003; Crozatier et al., 2004; Krzemień et al., 2007; Mandal et al., 2007). Notably, wasp parasitism elevates the reactive oxygen species (ROS) level in the PSC, which is important for cellular immune responses (see section “Role of the PSC in the Cellular Immune Response to Parasitic Wasp Infection”) (Crozatier et al., 2004; Sinenko et al., 2012; Louradour et al., 2017).

## Lymph Gland and the PSC

### Ontogeny and Terminal Differentiation of the Lymph Gland

The lymph gland is an important hematopoietic and immune organ of Drosophila. Ontogenetically, the development of the lymph gland starts at the early embryonic phase, and the naïve lymph gland forms at the late embryonic phase. At that time, a cluster of Col^+^Antp^+^ cells remain at the posterior of the lymph gland in the PSC, providing a niche for hematopoietic progenitors. Afterward, the lymph gland further develops, reaching maturity at the third-instar larval stage. Finally, dispersion of cells in the lymph gland occurs at the metamorphosis stage.

**FIGURE 1:**
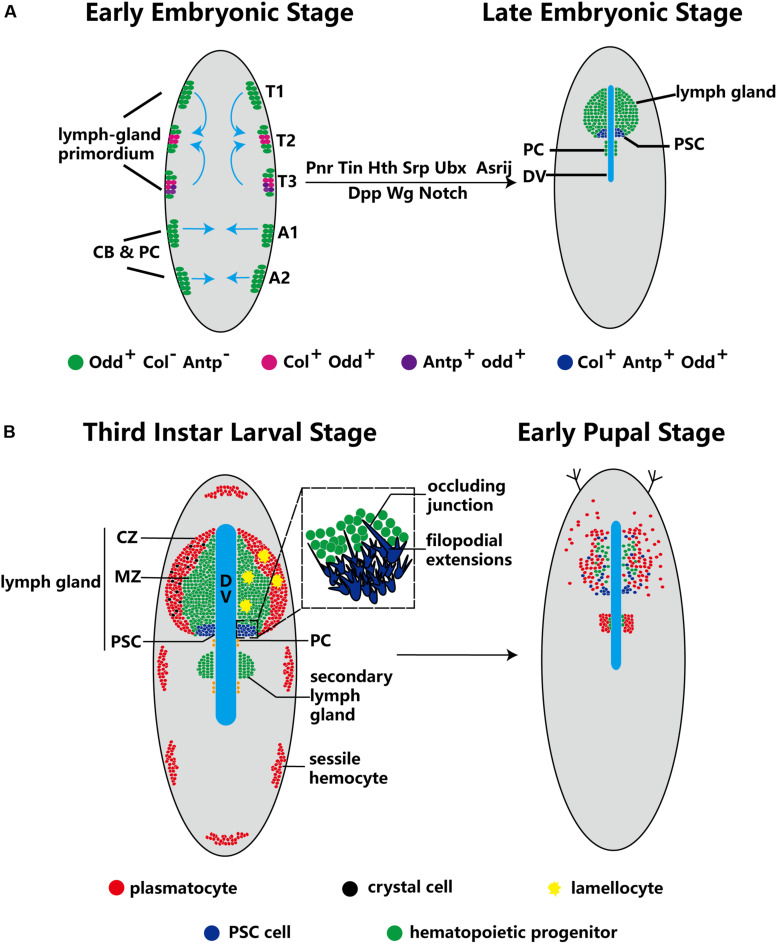
Ontogeny of the lymph gland and the PSC. **(A)** Odd^+^ cells form three pairs of cell clusters in the T1–T3 segments; the blue arrows indicate that these clusters coalesce to form the lymph gland. The PSC (purple) is defined by Col^+^ and Antp^+^ cells in the T2 and T3 segments. The progenitors, CBs and PCs, are closed lineages located in the cardiogenic mesoderm during the early embryo stage. At the late embryo stage, the lymph gland is along the dorsal vessel (DV), and PCs are located in the posterior lobes of the lymph gland. **(B)** In third-instar larvae, the lymph gland is mature and can be separated into three zones: the CZ, the MZ and the PSC. Three types of terminal mature hemocytes, plasmatocytes (red), crystal cells (black) and lamellocytes (yellow), are found in the lymph gland (lamellocytes rarely appear under normal conditions). The magnified view of PSC cells in the dotted-line box shows the occluding junction and filopodial extensions. During the early pupal stage, MZ cells show active proliferation and differentiation, and PSC cells are spread out. Subsequently, the lymph gland dissociates and releases hemocytes into the circulation.

At the early embryonic phase, lymph gland progenitors, cardioblast (CB) and pericardial (PC) cells, belong to a closed lineage and are located in the cardiogenic mesoderm (Figure 1A). Subsequently, the cardiogenic mesoderm gives rise to a part of the dorsal thoracic mesoderm and forms the origin of the lymph gland. The T1–T3 segments of the lateral thoracic mesoderm contain three pairs of cell clusters that express the transcription factor Odd-skipped (Odd) (Ward and Coulter, 2000; Mandal et al., 2007), and these cell clusters coalesce to form the lymph gland (Figure 1A; Mandal et al., 2004). Mutation of either the homeobox protein Tin or the GATA factor Pnr during this stage results in a missing lymph gland; moreover, the decapentaplegic (Dpp), heartless, Notch and wingless (Wg) pathways also participate in the formation of the lymph gland (Table 1; Mandal et al., 2004). The precursors of the PSC express Col and Antp (Mandal et al., 2007), and the separate cell clusters express Col in the T2 and T3 segments (Crozatier et al., 2004); however, Antp^+^ cells are located only in the T3 segment (Mandal et al., 2007). The cell clusters expressing either Col or Antp partially colocalize with cells expressing Odd and are progressively restricted to the posterior region before coalescing, at which point they become the PSC of the lymph gland in the larval stage. Other Odd^+^Col^–^Antp^–^ cells are precursors of hematopoietic progenitors that are triggered to mature via a dpp signal provided by the PSC at the first larval stage. These cells become the MZ or differentiate into mature hemocytes at the third larval stage (Figure 1A).

**TABLE 1:** Genes/pathways involved in the ontogeny of the lymph gland.

**Gene/pathway**	**Alteration**	**Phenotype**
Dpp	Loss of expression	Absence of lymph gland
Tin	Loss of expression	
Pnr	Loss of expression	
Hth	Loss of expression	
Wg	Loss of expression	
Notch	Loss of expression	Increased numbers of lymph gland cells
Srp	Overexpression	
Ubx	Loss of expression	
Asrij	Loss of expression	
Srp	Loss of expression	Transformation of the lymph gland primordium into pericardial cells
Ubx	Overexpression	
Hth	Overexpression	Reduced numbers of lymph gland PSC cells

Several studies have reported factors that influence lymph gland development. For example, loss of the GATA factor *serpent* (*srp*) causes the primordium to form pericardial cells but not lymph gland progenitors (Mandal et al., 2004). In addition, loss of the homeodomain cofactor *homothorax* (*hth*) causes failure of the formation of lymph glands; in contrast, overexpression of *hth* causes disappearance of the PSC (Mandal et al., 2007). On the other hand, loss of some genes causes lymph gland expansion. Several embryonic cell clusters express Ultrabithorax (Ubx), which can inhibit lymph gland formation. Upon loss of *Antp*, the lymph gland excessively expands into the abdominal segments at the early embryonic phase (Mandal et al., 2004). Furthermore, loss of the endocytic protein *Asrij* leads to excess lymph gland lobe formation at both the early embryonic and larval stages (Table 1; Kulkarni et al., 2011). These observations suggest that endosome trafficking is extremely important for lymph gland development.

During the metamorphosis stage of pupation, MZ cells actively proliferate and differentiate into terminal mature hemocytes, while PSC cells spread throughout the lymph gland (Grigorian et al., 2011). When the organism enters the pupal stage, the cells of the primary lobes of the lymph gland become large and transform into phagocytic cells. The lymph gland gradually dissociates, disappearing completely by 15 h after puparium formation, and releases hemocytes into the circulation (Figure 1B; Lanot et al., 2001; Grigorian et al., 2011).

### PSC Cell Features

Lebestky et al. (2003) found that the lymph gland contains a Ser-expressing signaling center on each primary lobe, which was defined as the PSC. Subsequently, Ser^+^ cells have also been detected in the CZ and posterior lobes of lymph glands (Ferguson and Martinez-Agosto, 2014). Crozatier et al. (2004) observed that Col, the *Drosophila* ortholog of the vertebrate gene encoding EBF, contributes to the differentiation of lamellocytes during the cellular immune response to parasitization. In addition, Ser has been found to colocalize with Col at the posterior tip of the lymph gland, and the levels of Ser are dependent on those of Col (Crozatier et al., 2004). Surprisingly, Col^+^ cells are not unique to the PSC but are also detected in the MZ at low levels (Benmimoun et al., 2015; Oyallon et al., 2016). These results have led to controversy regarding the localization of Col in the lymph gland (see section “Necessity of the PSC for Hematopoietic Progenitor Maintenance”). Mandal et al. (2007) showed that the PSC also expresses Antp during both the embryonic and larval stages. Moreover, the expression of Col is dependent upon that of Antp, indicating that Antp acts upstream of Col and Ser (Mandal et al., 2007).

At the early embryonic stage, the PSC of the lymph gland primordium can show active proliferation (Mandal et al., 2007). However, at the larval stage, PSC cells in the mature lymph gland are mitotically inactive (Lebestky et al., 2003; Mandal et al., 2007), and the number of PSC cells is maintained within the range of 30∼40. These data suggest that a robust mechanism that maintains stable numbers of PSC cells plays a key role in lymph gland homeostasis.

In addition, extensions of filopodia have been observed on PSC cells (Figure 1B). Knockdown of the EBF *col*, the GATA factor *srp*, the BAP chromatin-remodeling complex *osa*, and other important genes causes failure of filopodial extension in the PSC (Krzemień et al., 2007; Tokusumi et al., 2010, 2012); however, the detailed mechanisms have not been carefully characterized and require extensive further study. Khadilkar et al. reported the presence of occluding junctions between PSC cells. These occluding junctions establish a permeability barrier, the function of which is to regulate the communication of signals derived from the PSC to prohemocytes (hematopoietic progenitors) and to participate in the immune response (Khadilkar et al., 2017).

### Autonomous Regulation of PSC Cells

The number of PSC cells is stable at the third-instar larval stage, suggesting that autonomous or non-autonomous regulation prevents PSC cell proliferation (Table 2). In the 2000s, based on the initial definition of the PSC associated with the Notch ligand Ser, the Notch pathway was proposed to be a potential important regulator of the PSC. Early research published in 2004 indicated that the Col protein is required for the expression of Ser (Crozatier et al., 2004). However, research published in 2007 showed that the dominant-negative forms of both Ser and Notch cause the disappearance of Col-expressing cells (Krzemień et al., 2007). These studies indicate that there is an interdependence between Ser and Col expression in the regulation of PSC function.

**TABLE 2:** Genes/pathways involved in PSC cell regulation during the L3 stage.

**Gene/pathway**	**Cell type**	**Description**
dmyc	PSC cell	Promotes proliferation of PSC cells
Jumu	PSC cell	Regulates the number of PSC cells by promoting dmyc expression
Wg pathway		
E2F		
Dpp pathway	PSC cell	Regulates the number of PSC cells by inhibiting dmyc expression and acting as a putative wg inhibitor
Rbf	PSC cell	Regulates the number of PSC cells by inhibiting E2F expression
Bam	PSC cell	Regulates the number of PSC cells by promoting Rbf expression and inhibiting the putative E2F cooperator ElF4F
InR pathway	PSC cell	Responds to starvation, regulates the number of PSC cells by inhibiting translation regulation factor 4EBP expression and is regulated by feedback from dmyc through bantam
TOR pathway	PSC cell	Responds to starvation, regulates the number of PSC cells by inhibiting translation regulation factor 4EBP expression and engages in crosstalk with the InR pathway through Akt1
bantam	PSC cell	Regulates the number of PSC cells by promoting InR pathway signaling and is regulated by feedback from dmyc
ARF1	PSC cell	Regulates the number of PSC cells by affecting endosome trafficking through GTPase form switching to regulate PI3K activity in the InR pathway
Jumu	Progenitor	Regulates the number of PSC cells by promoting the expression of an unknown dmyc transcription inhibitor and by promoting PSC cell clustering through an unknown mechanism
ARF1	Mature hemocyte	Activates Asrij (as ARF1-GTP) and regulates the number of PSC cells through an unknown mechanism related to pvr
NA9	Mature hemocyte	Promotes proliferation of mature hemocytes and regulates the number of PSC cells through an unknown mechanism related to pvr
Slit/Robo	CT/PSC cell	Regulates the number of PSC cells by promoting Dpp pathway signaling and PSC cell clustering via modulation of DE-cad and Cdc42 activity

*Drosophila* Myc (dMyc), the ortholog of the mammalian protooncogene cMyc that encodes a transcriptional activator, regulates cellular growth and proliferation (Bouchard et al., 1998). Overexpression of dMyc in the PSC increases the number of PSC cells (to ∼100 cells) (Pennetier et al., 2012). Jumeau (Jumu) is a member of the forkhead (Fkh) transcription factor family and can bind the FKH domain of dMyc. Knockdown of *jumu* significantly decreases the number of PSC cells; in contrast, overexpression of *jumu* in the PSC significantly increases the number of PSC cells (Hao and Jin, 2017).

Both the Wg/Wnt and Dpp/bone morphogenetic protein (BMP) pathways are reported to be located upstream of dMyc (Gallant, 2009). Overexpression of *wg* increases the number of PSC cells; in contrast, expression of the inactive forms of the Wg pathway receptors Fz and DFz2 significantly reduces the number of PSC cells (Sinenko et al., 2009). Moreover, the increase in PSC cell number caused by overexpression of *wg* can be reversed by knockdown of *dmyc* (Pennetier et al., 2012). Therefore, the Wg/Wnt pathway indirectly regulates the proliferation of PSC cells by affecting dMyc levels. In contrast to Wg, Dpp/BMP limits the proliferation of PSC cells. Col downregulates dMyc by enhancing Dpp signaling, and knockdown of *col* significantly increases PSC cell numbers. Therefore, Col is also a self-autonomous negative regulator of PSC cells (Pennetier et al., 2012). In addition, depletion of the permeability barrier through knockdown of occluding junction components increases the number of PSC cells. When there is no permeability barrier, the strength of both Dpp and Wg signaling is decreased in PSC cells; however, the decrease in Dpp signaling overrides aberrant Wg levels and elevates dMyc expression (Khadilkar et al., 2017). These observations suggest that permeability contributes to the regulation of PSC cell numbers through Dpp and Wg signaling.

The *Drosophila* insulin/insulin-like growth factor signaling (IIS) pathway is related to metabolism, translation and protein synthesis (Sousa-Nunes et al., 2011; Tennessen and Thummel, 2011). The target of rapamycin (TOR) pathway is another protein synthesis-regulating signaling pathway that engages in crosstalk with the IIS pathway through Akt1. Both the IIS and TOR pathways have been shown to be involved in dMyc expression and regulation, suggesting that both pathways and their related factors are candidates for autonomous regulation of PSC cells. As expected, increased IIS signaling in the PSC through activation of phosphoinositide-3 kinase (PI3K), loss of FoxO or phosphatase and tensin homolog (PTEN), and overexpression of the insulin receptor (InR) or Akt1 can induce PSC expansion (Benmimoun et al., 2012; Tokusumi et al., 2012). Notably, vesicle trafficking is important for signal transduction. ADP ribosylation factor 1 (ARF1) is required for coat assembly, the Golgi architecture and endosome trafficking. ARF1 balances the IIS pathway in PSC cells by partially affecting PI3K. Furthermore, ARF1 can regulate Asrij and participate in lymph gland development by switching between GDP- and GTP-binding forms. Low levels of ARF1 in the PSC can inactivate Asrij and cause complete loss of the PSC (Khadilkar et al., 2014). In addition, Bantam miRNA regulates IIS signaling by affecting Akt1 and is activated by dMyc. Loss of *bantam* significantly reduces the number of PSC cells; however, this effect can be reversed by overexpression of *dmyc*. Moreover, the increase in PSC cell number caused by overexpression of *bantam* can be reversed by knockdown of the InR pathway (Lam et al., 2014). Therefore, dMyc is located between Bantam and the InR pathway and regulates the signaling cascade and feedback loop.

eIF4E (4E)-binding protein (4EBP), which is an inhibitor of the elF4F complex, is inhibited by the IIS pathway (Tokusumi et al., 2011, 2012). Furthermore, Bag of Marbles (Bam), a negative regulator of the elF4F complex, inhibits the proliferation of PSC cells (Tokusumi et al., 2011). Retinoblastoma-family protein (Rbf), a suppressor of cell proliferation regulated by the Bam protein, can suppress the levels of E2F, one of several transcription factors that regulate dMyc levels. Accordingly, Rbf inhibits the proliferation of PSC cells (Tokusumi et al., 2015). These results indicate that the IIS pathway may regulate dMyc through both Bantam and the regulatory relationship between the F4F complex and E2F; however, the mechanisms involved in these interactions and the regulation of PSC development need to be further investigated.

**FIGURE 2:**
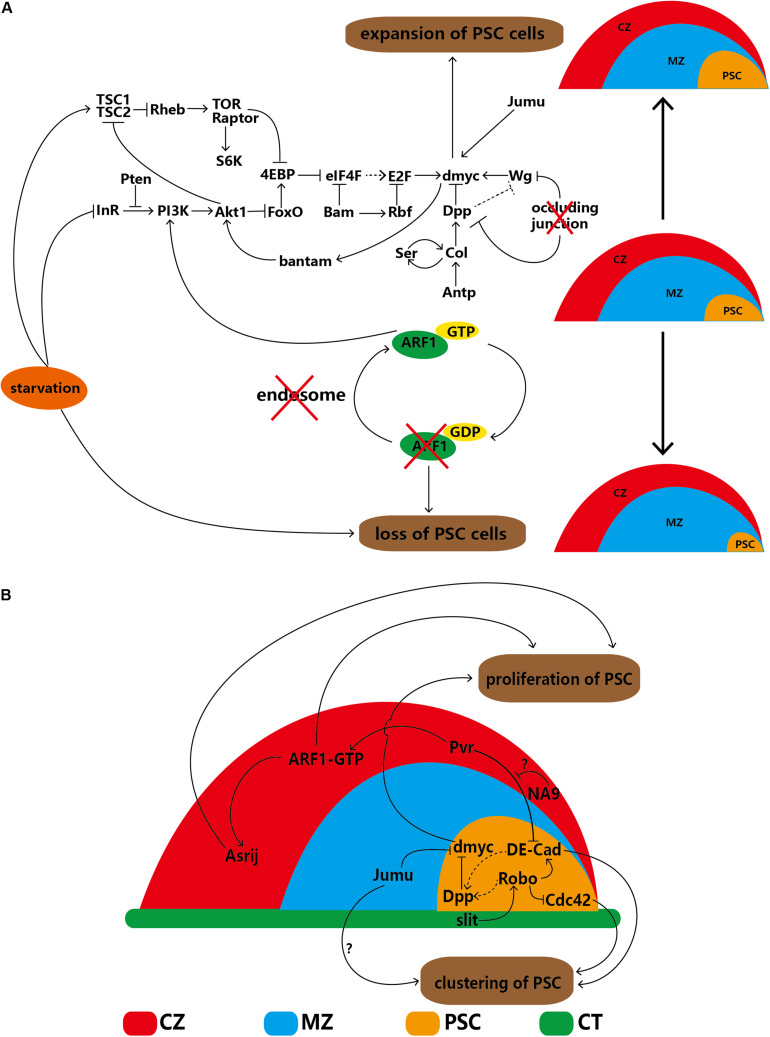
Regulatory network of PSC cells in the lymph gland. **(A)** PSC cells are self-autonomously regulated by several factors that lead to increases or decreases in cell number. **(B)** The proliferation and clustering of PSC cells are regulated by extrinsic factors of the CZ, MZ and DV. The solid arrows indicate positive regulation, the ‘T’-shaped arrows indicate negative regulation, the dotted arrows indicate a putative regulatory relationship, the red cross indicates that the component below is disturbed, and the question marks (‘?’) indicate that the detailed regulatory mechanism is uncertain. CZ, cortical zone; MZ, medullary zone; CT, cardiac tube.

Enhancement of TOR signaling through knockdown of tuberous sclerosis complex 2 (TSC2) causes PSC expansion; in contrast, attenuation of TOR signaling through knockdown of *rictor*, *raptor*, *Tor*, *Rheb* or *S6K* reduces PSC cell numbers. In addition, 4EBP is a downstream target of both the IIS and TOR pathways; therefore, the TOR pathway regulates PSC cells by inhibiting 4EBP. Both the IIS and TOR pathways are related to nutrient status. As expected, starvation decreases IIS and TOR pathway signaling and induces loss of the PSC (Shim et al., 2012, 2013; Tokusumi et al., 2012). Therefore, the IIS and TOR pathways, along with their related factors and dMyc, establish a complex interdependent regulatory network. In summary, dMyc, a hub gene, can autonomously regulate the network of PSC cells, as illustrated in Figure 2A.

### Non-autonomous Regulation of PSC Cells

In-depth study in recent years has revealed that some cells adjacent to the PSC can regulate the proliferation or clustering of PSC cells, indicating that non-autonomous regulation exists. Cardiac tube (CT) cells and the slit/Roundabout (Robo) pathway were first discovered because the PSC is located alongside the CT. Robo, the receptor of Slit, can be activated by Slit, which is derived from the CT. Activated Robo promotes Dpp pathway signaling directly and indirectly via DE-cadherin (DE-Cad), thereby negatively regulating the proliferation of PSC cells. Under normal conditions, the number of PSC cell clusters is maintained through the dual functions of DE-Cad and Cdc42. Knockdown of *Robo* in the PSC significantly increases the number of PSC cells and splits the cells into several cell clusters; knockdown of *slit* in the CT results in an identical phenotype (Morin-Poulard et al., 2016), indicating the existence of communication between the CT and lymph gland (Figure 2B).

Knockdown of *jumu* in MZ cells elevates dMyc levels in the PSC through an unknown mechanism, not only significantly increasing PSC cell numbers but also splitting PSC cells into several clusters. However, the DE-Cad and Cdc42 levels remain unchanged (Hao and Jin, 2017). In the CZ, specific knockdown of the ARF1 guanine nucleotide exchange factor (Gartenzwerg) or overexpression of a GTPase-activating protein that keeps Gartenzwerg in its inactive form results in significant loss of PSC cells (Khadilkar et al., 2014). In addition, overexpression of NUP98-HOXA9 (NA9) causes significant expansion of PSC cells, and the candidate mechanism involves signaling mediated by platelet-derived growth factor (PDGF)/vascular endothelial growth factor (VEGF)-related receptor (Pvr) from mature hemocytes to DE-Cad in PSC cells. This observation reinforces Pvr as a regulator of PSC cell numbers and clustering. However, NA9 in the CZ cannot regulate Pvr expression directly (Baril et al., 2017). The differentiated cells of the CZ can control progenitors, which are triggered by binding of PDGF/VEGF-related factor 1 (Pvf1) of the PSC to Pvr on CZ cells (Mondal et al., 2011) (see details in section “The PSC Indirectly Maintains the Homeostasis of Progenitors via Hemocyte Differentiation”). Both ARF1 and NA9 in the CZ interact with Pvr (Baril et al., 2017); however, the detailed mechanism by which Pvr regulates DE-Cad in PSC cells requires clarification (Figure 2B).

## The Psc Functions as a Hematopoietic Progenitor Niche

Undoubtedly, the balance between hematopoietic progenitor maintenance and differentiation is very important. PSC cells, which compose a hematopoietic progenitor niche, contribute to maintaining this balance via direct and indirect maintenance of progenitor regulation. In addition, PSC cells are able to convert hematopoietic progenitor precursors into mature hematopoietic progenitors at the first larval stage. However, two studies in 2015 and 2016 sparked controversy regarding the necessity of PSC cells for the maintenance of hematopoietic progenitors in the MZ.

### The PSC Directly Maintains the Homeostasis of Hematopoietic Progenitors

**FIGURE 3:**
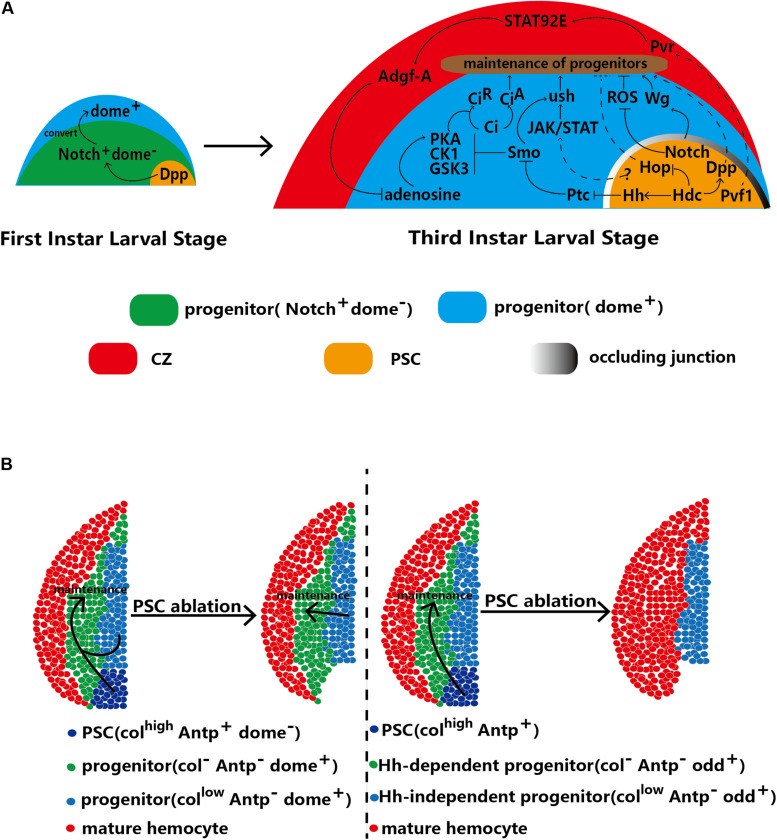
The PSC regulates the homeostasis of hematopoietic progenitors. **(A)** In first-instar larvae, the PSC regulates the conversion of Notch^+^/Dome^–^ progenitors into Dome^+^ progenitors. At the third larval stage, the PSC maintains the homeostasis of hematopoietic progenitors through direct and indirect mechanisms. **(B)** Two contradictory models with differing views on the requirement of the PSC for hematopoietic progenitor maintenance. The left model indicates that the PSC (defined by Antp^+^ cells) is redundant and that progenitors with low Col expression are sufficient for the maintenance of hematopoietic progenitors. The right model indicates that Hh-dependent (Odd^+^ Col^–^) progenitors are maintained by the PSC. Upon ablation of the PSC, the progenitors in the subset differentiate into mature hemocytes, which results in a decrease in MZ size. Other Hh-independent progenitors are kept quiescent by autonomous maintenance.

PSC cells can secrete ligands directly into the MZ, which activates a corresponding signaling pathway related to the maintenance of hematopoietic progenitors in the activated MZ. The Hedgehog (Hh) signaling pathway is related to cell growth and differentiation (Lum and Beachy, 2004). Hh signaling has been widely studied in the *Drosophila* lymph gland and is viewed as a major maintenance process of the hematopoietic progenitor pathway directly activated by the PSC because all PSC cells express the ligand Hh. Loss of Hh signaling promotes hematopoietic progenitor differentiation into plasmatocytes and crystal cells; in contrast, increased Hh secretion from the PSC inhibits the differentiation of lamellocytes (Mandal et al., 2007). The progenitors of the MZ have two receptors: Patched (Ptc) and Smoothened (Smo) (Mandal et al., 2007; Giordani et al., 2016; Baldeosingh et al., 2018). In the absence of Hh, Ptc and Smo mediate Cubitus interruptus (Ci) in a repressor form (Ci^R^) via phosphorylation and degradation (Akimaru et al., 1997; Aza-Blanc et al., 1997; Chen et al., 1999). In contrast, the PSC-secreted Hh protein can be transferred to the MZ and bind with Ptc to trigger the Hh signaling pathway in the lymph gland. Inactivation of Ptc can release the receptor Smo (Mandal et al., 2007; Tokusumi et al., 2018) and prevent Ci phosphorylation and degradation by protein kinase complexes of protein kinase A (PKA), casein kinase 1 (CK1) and glycogen synthase kinase-3 (GSK3) to maintain Ci in its activated form (Ci^A^), thereby maintaining hematopoietic progenitors (Figure 3A; Chen et al., 1998; Price and Kalderon, 2002; Lum et al., 2003; Zhang et al., 2006).

Notably, Hh signaling is not induced until 18 h after egg hatching (AEH). This timing implies that before 18 h AEH, other signaling pathways play a role similar to that of the Hh pathway. There are no mature hemocytes in the lymph glands of early first-instar larvae (13 h AEH); only two types of hematopoietic progenitors, Notch^+^/domeless (Dome)^–^ and Notch^–^/Dome^+^ cells, exist. Recently, PSC-secreted Dpp has been suggested to potentially play a role in progenitor maintenance. Dpp is a ligand of the BMP pathway that is secreted from PSC cells and contributes to converting early progenitors (progenitor precursors) into mature progenitors. For example, Dpp promotes the conversion of Notch^+^/Dome^–^ progenitors into Dome^+^ progenitors at 30 h AEH. Then, these Dome^+^ progenitors form the MZ of the lymph gland at the third-instar larval stage. Weak Dpp secretion from PSC cells of first-instar larvae causes loss of Dome^+^ progenitors in the lymph gland at the third-instar stage (Dey et al., 2016; Figure 3A). In addition, knockdown of Headcase (Hdc), the homolog of the tumor suppressor HECA, in the PSC has been shown to enhance Dpp secretion from PSC cells and inhibit lamellocyte differentiation (Varga et al., 2019).

In the second and third larval stages, the Hh and Antp proteins are specifically expressed only in the PSC (Mandal et al., 2007). In general, the number of PSC cells reflects the strength of Hh signaling (Mandal et al., 2007; Benmimoun et al., 2012; Lam et al., 2014; Tokusumi et al., 2015), and an increase in PSC cell number strongly inhibits the differentiation of progenitors caused by high levels of Hh. However, some studies have produced results inconsistent with these findings. For example, blockade of occluding junctions in the PSC has been found to promote the proliferation of PSC cells but not to increase the levels of Hh, which can promote the differentiation of progenitors (Khadilkar et al., 2017). Col has been found to inhibit the proliferation of PSC cells; however, low levels of Col increase PSC cell numbers without inhibiting the differentiation of progenitors and decrease MZ size (Pennetier et al., 2012). The GATA factor Srp is a positive regulator of Hh signaling; however, knockdown of *srp* specifically in the PSC does not change PSC cell numbers (Tokusumi et al., 2010). Similarly, PSC-specific overexpression of *Drosophila* friend of GATA transcriptional regulator, U-shaped (*ush*), a negative regulator of Hh, has no effect on PSC cell numbers.

In addition to PSC-secreted Hh and Dpp, aberrant PSC impacts the maintenance of hematopoietic progenitors signaling. The Janus kinase (JAK)/signal transducer and activator of transcription (STAT) receptor Dome is regarded as a marker in hematopoietic progenitors of the MZ. JAK/STAT signaling maintains hematopoietic progenitors via Ush (Gao H. et al., 2009; Tokusumi et al., 2010). Moreover, Ush is regulated by Smo, a receptor of the Hh pathway (Baldeosingh et al., 2018). In addition, Krzemień et al. observed that PSC cells activate JAK/STAT signaling in the MZ. Loss of Col expression induces PSC cell loss and reduces Dome^+^ cell numbers in the MZ (Krzemień et al., 2007; Pennetier et al., 2012). In summary, the PSC, JAK/STAT pathway and Ush constitute a regulatory network that controls the differentiation of hematopoietic progenitors (Figure 3A). In addition, the JAK/STAT pathway in PSC cells regulates the differentiation of progenitors via an unknown mechanism. Knockdown of *hdc* induces lamellocyte differentiation, which can be reversed through knockdown of *hop*, a kinase for ligands of the JAK/STAT pathway in PSC cells (Varga et al., 2019). However, whether the JAK/STAT pathway in PSC cells contributes to the differentiation of MZ cells by affecting the JAK/STAT levels of progenitors needs to be further examined.

Wg signals also maintain hematopoietic progenitors (Sinenko et al., 2009). The permeability barrier consists of occluding junctions between PSC cells, which suggests that the Wg levels of PSC cells can extend to progenitors. Depletion of the permeability barrier reduces Wg signaling in both PSC cells and progenitors, thereby promoting the differentiation of progenitors (Khadilkar et al., 2017).

Notch signaling in hematopoietic progenitors promotes the differentiation of crystal cells (Ferguson and Martinez-Agosto, 2014). In addition, the Notch ligand Ser is regarded as another marker of PSC cells (Lebestky et al., 2003; Yu et al., 2018), and loss of *ser* decreases PSC cell numbers and promotes the differentiation of progenitors (Krzemień et al., 2007). However, recent studies have shown that there is no relationship between the strength of the Notch signal in the PSC and that in the MZ or CZ of the lymph gland (Small et al., 2014). Therefore, communication between the PSC and MZ through Notch signaling needs to be further defined. In 2009, ROS caused by oxidative stress were proven to promote differentiation of hematopoietic progenitors in the MZ (Owusu-Ansah and Banerjee, 2009). Small et al. showed that ROS in the MZ are inhibited by Notch signals of the PSC through an unknown pathway. Knockdown of the Notch signal in PSC cells causes high levels of ROS to accumulate in the MZ and numerous lamellocytes (Figure 3A; Small et al., 2014).

### The PSC Indirectly Maintains the Homeostasis of Progenitors via Hemocyte Differentiation

Pvf1 originates from the PSC and is transported into the CZ, which contains Pvr (Mondal et al., 2011). Binding of Pvf1 to Pvr in the CZ results in a signaling cascade between the CZ and MZ in lymph glands. STAT92E is activated by Pvr and induces overexpression of adenosine deaminase-related growth factor A (Adgf-A). Next, Adgf-A downregulates adenosine in adjacent MZ cells, the function of which is to promote PKA to phosphorylate and degrade the key Hh signaling pathway component Ci into the active form Ci^A^ (Chen et al., 1998; Mondal et al., 2011), which contributes to the maintenance of hematopoietic progenitors. This cross-region regulatory pathway, called the equilibrium signaling pathway, is analogous to the Hh signaling pathway (Figure 3A; Mondal et al., 2011, 2014; Ferguson and Martinez-Agosto, 2017).

### Necessity of the PSC for Hematopoietic Progenitor Maintenance

The PSC has traditionally been thought to be required for the maintenance of hematopoietic progenitors. However, in 2015, Benmimoun et al. raised doubts about the previous understanding of the relationship between the MZ and PSC (Crozatier et al., 2004; Benmimoun et al., 2015). These researchers found that a low level of Col expression in the MZ functioned similarly to that in the PSC by inhibiting the differentiation of hematopoietic progenitors. Furthermore, Col expression in the MZ and Col expression in the PSC were found to be independent. Antp is specifically expressed in the PSC at the third-instar larval stage. Surprisingly, neither ablation of *antp* through null mutation nor apoptosis of PSC cells driven by the proapoptotic protein Reaper (rpr) decreases the numbers of prohemocytes (cells in the MZ marked by Dome-GFP and Tep4). However, *col* null mutation promotes the differentiation of progenitors (Benmimoun et al., 2015). A follow-up study has also confirmed that PSC cells are not required for the maintenance of progenitors. Decreasing the number of PSC cells by knocking down Wg signaling or inhibiting the Hh signaling pathway of the PSC by knocking down Srp does not induce prohemocyte differentiation (Figure 3B; Oyallon et al., 2016). In this model, Col in MZ cells is sufficient for the maintenance of hematopoietic progenitors, and PSC cells appear redundant. Therefore, the traditional model in which the PSC functions to regulate hematopoietic progenitors has been questioned.

More recent studies have further confused the matter, leading to controversy. In 2018, Baldeosingh et al. examined the effects of specific ablation of PSC cells driven by rpr; however, their results contradicted those of the previous study. They analyzed the colocalization of Col and Odd in the MZ upon ablation of the PSC and found that the Col^–^Odd^+^ subpopulation of prohemocytes differentiated into mature hemocytes; however, other Odd^+^ prohemocytes expressing low levels of Col were maintained (Baldeosingh et al., 2018; Figure 3B). Sharma et al. (2019) split the MZ into Hh-independent and Hh-dependent progenitors, supporting this finding. These results may be more trustworthy than the former because prohemocytes were detected with various markers, such as Odd, E-cadherin, Dome-MESO-LacZ and Ci (Jung et al., 2005; Yu et al., 2018). In addition, two studies have shown that the Hh protein derived from PSC cells is a key component that regulates hematopoietic homeostasis (Mandal et al., 2007; Tokusumi et al., 2018). The potential redundancy of the PSC in the maintenance of progenitors calls into question the functions of signaling pathways derived from the PSC. However, to date, no alternative mechanism to the signaling pathways of the PSC has been defined. In this model, hematopoietic progenitors are classified into two subpopulations: Col-low and Col-negative progenitors. However, only Col-negative progenitors are required for PSC maintenance.

In summary, studies examining the regulatory functions of PSC cells have yielded several contradictory results; therefore, this topic requires deeper investigation. Interestingly, all of the studies discussed here have consistently shown that PSC cells are required for lamellocyte differentiation in response to wasp parasitism. When the PSC is ablated, progenitors fail to differentiate into lamellocytes in response to wasp parasitism (Crozatier et al., 2004; Krzemień et al., 2007; Benmimoun et al., 2015).

## Role of the Psc in the Cellular Immune Response to Parasitic Wasp Infection

The parasitoid wasp *Leptopilina boulardi* is commonly used to infect fruit flies in order to study the cellular immune response to invasion (Carton and Nappi, 2001). The dedicated cellular immune response promotes the proliferation of hemocytes and induces massive differentiation into lamellocytes, whose function is to encapsulate wasp eggs (Lanot et al., 2001). At the end of infection, the lymph gland is disrupted and releases many lamellocytes (Krzemień et al., 2007). Notably, the PSC is more sensitive to wasp infection than other regions; the Toll/nuclear factor κB (NFκB) immune signaling pathway is activated in PSC cells even under normal conditions (Gueguen et al., 2013), suggesting that the high Toll/NFκB levels of the PSC allow this region to be constantly prepared to resist environmental infection. As described above, PSC undoubtedly plays a key role in the cellular immune response to parasitic wasps.

**FIGURE 4:**
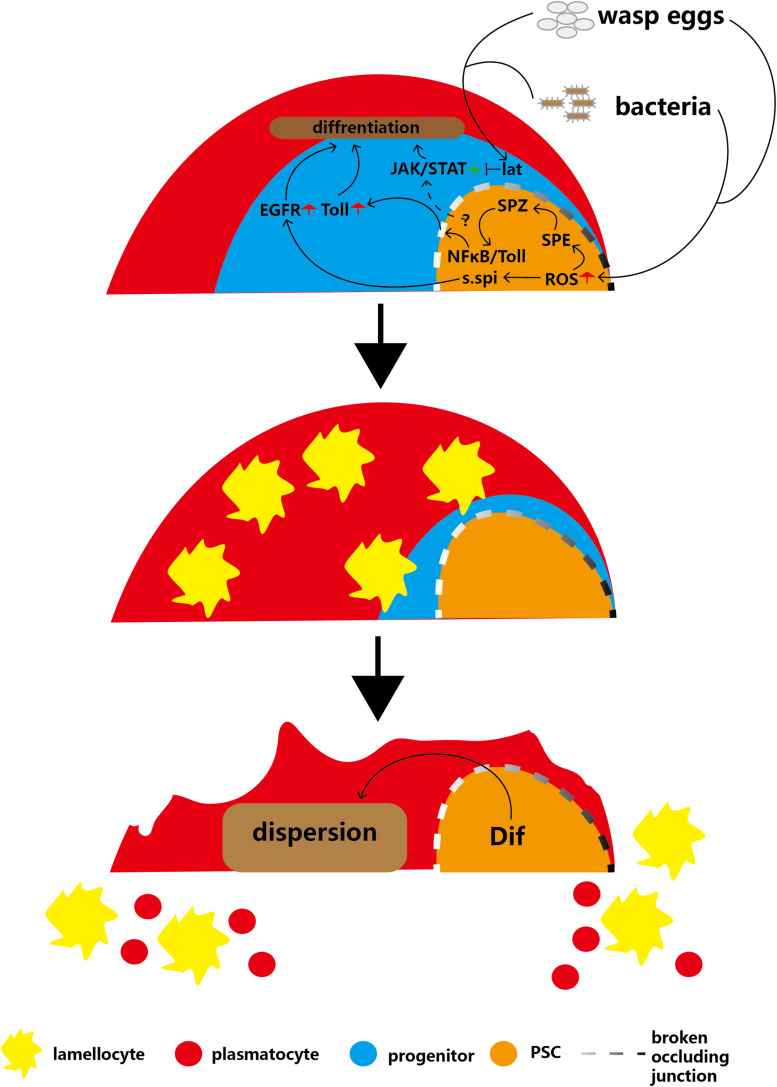
Infection with parasitic wasps or bacteria induces production of lamellocytes and disruption of the lymph gland. Parasitic wasp or bacterial infection induces a decrease in MZ size or an increase in ROS levels in the PSC, respectively, and causes activation of the EGFR and Toll pathways in the MZ. In addition, infections inhibit the JAK/STAT pathway through Lat, which overrides PSC maintenance JAK/STAT levels. These two pathways cause massive differentiation of lamellocytes. The Dif-dependent Toll/NFκB pathway contributes to the disruption of the lymph gland to release mature hemocytes into circulation.

Wasp infection causes high levels of ROS to accumulate in the PSC, which can trigger the secretion of Spitz (s.spi), a ligand for epidermal growth factor receptor (EGFR), into the MZ, subsequently activating the EGFR signaling pathway of anterior lobe cells and causing massive lamellocyte differentiation in the lymph glands (Sinenko et al., 2012; Figure 4). In 2017, follow-up research showed that another signaling pathway triggered by high ROS levels in PSC cells contributes to the immune response to wasp infection (Louradour et al., 2017). ROS can promote cleavage of the Toll/NFκB pathway ligand Spätzle (Spz) by Spätzle-processing enzyme (SPE), therefore activating the Toll/NFκB pathway in the PSC (Jang et al., 2006). The activated NFκB pathway of the PSC induces breakdown of the permeability barrier (made of occluding junctions), which enables the immune signal to extend into the MZ (Khadilkar et al., 2017). Next, the Toll/NF-kB pathway of the MZ is activated and promotes the differentiation of lamellocytes. Finally, high levels of Dif and Toll pathway transcription factors contribute to the dispersion of mature hemocytes from the lymph gland into the circulation. These findings suggest that the EGFR and Toll/NFκB pathways may act in parallel in response to wasp infection in PSC cells to regulate lamellocyte differentiation. However, although the EGFR and Toll/NFκB pathways have similar functions and triggering mechanisms in hematopoiesis, the relationship between the two pathways is unclear (Sinenko et al., 2012; Louradour et al., 2017; Figure 4).

In addition, PSC cells maintain JAK/STAT pathway signaling in hematopoietic progenitors to inhibit prohemocyte differentiation (Krzemień et al., 2007; Gao H. et al., 2009). After wasp infection, the massive prohemocyte differentiation into mature hemocytes suggests that JAK/STAT signaling in progenitors is deactivated because PSC cells lose the capacity to maintain the JAK/STAT pathway. In addition, a short type I cytokine-related receptor, latran (lat), is activated by wasp infection. Next, activated lat in hematopoietic progenitors inhibits the JAK/STAT pathway to promote the differentiation of prohemocytes into lamellocytes (Figure 4; Makki et al., 2010). This indicates that after wasp infection, inhibition of lat can override the PSC cell-mediated maintenance of the hematopoietic progenitor JAK/STAT pathway.

## Psc of the *Drosophila* Lymph Gland as a Model for Investigating the Mammalian Bone Marrow Hematopoietic Stem Cell Niche

### Similar HSC Niche Regulatory Mechanisms in *Drosophila* and Mammals

Mammalian hematopoietic stem cells (HSCs), which form the basis for mammalian hematopoietic processes, have the capability to produce all mature blood cell lineages. In *Drosophila*, the development of HSCs occurs in two main waves: the embryonic stage and the adult stage. During mammalian embryogenesis, HSC precursors migrate through different embryonic niches to develop into mature HSCs, which is reminiscent of the function of the *Drosophila* lymph gland PSC at the first larval stage (Mikkola and Orkin, 2006; Dey et al., 2016) (see section “The PSC Directly Maintains the Homeostasis of Hematopoietic Progenitors”). In adults, the main HSCs reside in the bone marrow niche. An HSC niche can be defined as a location from which housing and maintenance regulatory signals are derived. HSC niche regulatory signals cause HSCs to remain quiescent under normal conditions and to differentiate or proliferate during immune responses (Suda et al., 2005; Pinho and Frenette, 2019).

Two types of mammalian HSC niches have been identified: the endosteal niche and the perivascular niche. HSCs are attached to osteoblasts of trabecular bone in the endosteal niche and to the fenestrated endothelia of bone marrow sinusoids in the perivascular niche (Wilson and Trumpp, 2006; Kiel and Morrison, 2008; Celso et al., 2009; Xie et al., 2009). Two long-term problems in the field of mammalian HSC research are the difficulty in dissecting niches located at various anatomical sites and the lack of specific markers for identification of hematopoietic niches (Mikkola and Orkin, 2006; Pinho and Frenette, 2019). Compared to mammalian niches, the PSC of the *Drosophila* lymph gland is a unique HSC niche and expresses several specific markers (see section “PSC Cell Features”). In summary, the *Drosophila* PSC is an ideal model for investigating the mammalian HSC niche regulatory mechanism.

As described above, the Hh, Dpp/BMP, wingless, Notch and Slit/Robo signaling pathways regulate hematopoietic progenitors via the PSC. Hh signaling has been conserved throughout evolution, including in mammalian HSCs (Gao J. et al., 2009; Hofmann et al., 2009). Moreover, loss of BMP receptor 1A (BMPR1A) in the mouse bone marrow stroma increases the numbers of both osteoblasts and repopulating HSCs; this effect is similar to the phenotype induced by loss of the BMP signal in the *Drosophila* PSC (Zhang et al., 2003; Pennetier et al., 2012). Wnt in mammals is analogous to Wg in *Drosophila* (Siegfried and Perrimon, 1994; Cavallo et al., 1998; Packard et al., 2002). Overexpression of Wg in PSC cells promotes PSC cell proliferation and blocks hematopoietic progenitor differentiation (Sinenko et al., 2009; Pennetier et al., 2012), and similar observations have been made in mammals. Overexpression of Ctnnb1, a primary regulatory target of Wnt signaling, increases the HSC pool by blocking HSC differentiation (Kirstetter et al., 2006; Scheller et al., 2006). Notch also participates in developmental signaling pathways. The *Drosophila* PSC can be defined by a cell cluster expressing the Notch ligand Ser (Lebestky et al., 2003). Similarly, mammalian HSC niche osteoblasts express Notch1 and Notch3 and the ligands Jagged1 and Dll1 (Li et al., 1998; Stier et al., 2002; Dando et al., 2005). In addition, the Notch pathway in both mammalian HSC niches and the *Drosophila* PSC can affect hematopoietic differentiation. However, the detailed regulatory mechanism in the mammalian HSC niche, like that in the *Drosophila* PSC, remains unclear (Renström et al., 2010; Small et al., 2014). In *Drosophila*, Slit, secreted by the CT, binds to its receptor Robo in the PSC to activate the Slit/Robo signaling pathway (Morin-Poulard et al., 2016). Similarly, slit2-Robo4 regulates HSC localization in the bone marrow perivascular niche (Smith-Berdan et al., 2011, 2012). During infection, Toll-like receptors are expressed by mammalian HSCs and can sense pathogens. Upon activation, NFκB/Toll induces HSCs to differentiate into immune cells (Nagai et al., 2006; Zhao et al., 2014). Analogous to the case in mammals, elevations in ROS levels in *Drosophila* PSC cells caused by pathogen infection can activate NFκB/Toll signaling in hematopoietic progenitors and promote the differentiation of plasmatocytes and lamellocytes (see section “Role of the PSC in the Cellular Immune Response to Parasitic Wasp Infection”).

However, there are still some regulatory mechanisms of niche HSCs that are specific to either *Drosophila* or mammals. In mammals, loss of membrane-bound stem cell factor (SCF) decreases the numbers of osteoblasts and HSCs (McCulloch et al., 1965; Lyman and Jacobsen, 1998), and knockout of core-binding factor subunit α1 (CBFα1) causes complete loss of osteoblasts and HSCs (Komori et al., 1997; Otto et al., 1997; Deguchi et al., 1999). Although the *Drosophila* ortholog lozenge (lz) participates in crystal cell differentiation, there is no evidence that lz contributes to the regulation of niche HSCs (Evans et al., 2003). Constitutive activation of parathyroid hormone (PTH) or the PTH/PTH-related protein receptor (PPR) specifically in osteoblasts can increase both osteoblast and HSC populations in mouse bone marrow (Calvi et al., 2003). Furthermore, Cxcl12 and its receptor Cxcr4 have been shown to contribute to niche HSC regulation (Zou et al., 1998; Sugiyama et al., 2006; Tzeng et al., 2011).

### *Drosophila* PSC and Leukemia

Leukemia is a kind of malignant disease whose symptoms include increased numbers of leucocytes in the blood or bone marrow. As observed in human leukemia, melanotic tumors, which are composed of aggregated hemocytes and large numbers of abnormal lamellocytes, can appear in *Drosophila* (Boulet et al., 2018). Leukemic stem/progenitor cells (LSCs) or leukemia-initiating cells (LICs) are the root mediators of leukemia initiation, drug resistance and relapse (Zhou and Xu, 2015; Pulikkan and Castilla, 2018). Reducing the maintenance of LSCs and LICs through niche regulatory signaling is a therapeutic strategy. The genetically conserved Hh pathway has been demonstrated to be required for leukemia-initiating cell differentiation (Dagklis et al., 2015; Lim et al., 2015), and *Drosophila* Hh signaling is derived from PSC cells (Mandal et al., 2007). In PSC cells, Hh signaling inhibits Ush expression, and direct knockdown of Hh signaling via Hh RNAi can result in a leukemia-like phenotype (Mandal et al., 2007; Baldeosingh et al., 2018). Furthermore, Hh signaling pathway inhibitors can be applied for clinical therapy of Bcr-Abl leukemia (Helgason et al., 2010). Feeding of PF-04449913, an inhibitor of the human Hh pathway receptor Smo, to *Drosophila* larvae significantly promotes LSC differentiation and blocks relapse (Giordani et al., 2016).

In mammals, LSCs can modulate and hijack the hematopoietic niche to outcompete normal HSCs (Nwajei and Konopleva, 2013; Pinho and Frenette, 2019), and similar capabilities have been observed in *Drosophila*. Notably, HOX transcription factors have been observed to be dysregulated in acute myeloid leukemia (AML). Misexpression of NA9 in mature *Drosophila* hemocytes using the UAS/Gal4 system not only promotes hemocyte proliferation and hyperplastic growth of the lymph gland but also induces PSC expansion (Baril et al., 2017). In addition, misexpression of NA9 can interfere with Pvr signaling, which is involved in leukemia (Stamm et al., 2018; Hattori et al., 2019). These observations suggest that proliferation of PSC cells is related to the leukemia-like phenotype.

## Conclusion and Perspectives

The *Drosophila* lymph gland is a powerful model for studying hematopoiesis, and the PSC has become an ideal model for studying niche interactions with HSCs. However, some questions about the PSC remain to be answered. For example, whether the PSC is required for the maintenance of hematopoietic progenitors or whether Col-positive progenitors are sufficient for this maintenance remains an important question. In addition, whether the number of PSC cells represents the strength of the Hh signaling pathway is unclear. Furthermore, the detailed functions of filopodial extensions of PSC cells have yet to be elucidated. Finally, although the PSC is undoubtedly the unique niche of hematopoietic progenitors in the lymph gland, hematopoietic progenitors are very rarely found among circulating hemocytes (Osman et al., 2009; Sinenko et al., 2010); thus, whether some cells provide a niche for circulating hematopoietic progenitors deserves to be studied.

Overall, we have summarized several important points regarding the *Drosophila* lymph gland PSC: (1) that autonomous and non-autonomous regulatory networks maintain stable numbers of PSC cells; (2) that PSC cells regulate the homeostasis of hematopoietic progenitors not only directly but also indirectly via mature hemocyte equilibrium signals; (3) that the requirement of the PSC for the maintenance of hematopoietic progenitors is currently controversial; (4) that PSC cells play a key role in the immune response via elevations in ROS levels; and (5) that the *Drosophila* lymph gland PSC can be used to investigate the relationship between leukemia and the mammalian bone marrow hematopoietic niche.

## Author Contributions

FL and SY contributed to the writing of this review article. LJ approved the final version of the manuscript. FL, SY, and LJ are accountable for the entire contents.

## Conflict of Interest

The authors declare that the research was conducted in the absence of any commercial or financial relationships that could be construed as a potential conflict of interest.
